# How we provided appropriate breast imaging practices in the epicentre of the COVID-19 outbreak in Italy

**DOI:** 10.1259/bjr.20200679

**Published:** 2020-09-02

**Authors:** Filippo Pesapane, Silvia Penco, Anna Rotili, Luca Nicosia, Anna Bozzini, Chiara Trentin, Valeria Dominelli, Francesca Priolo, Mariagiorgia Farina, Irene Marinucci, Stefano Meroni, Francesca Abbate, Lorenza Meneghetti, Antuono Latronico, Maria Pizzamiglio, Enrico Cassano

**Affiliations:** 1 Breast Imaging Division, IEO European Institute of Oncology IRCCS, Milan, Italy

## Abstract

Italy has one of the highest COVID-19 clinical burdens in the world and Lombardy region accounts for more than half of the deaths of the country. Since COVID-19 is a novel disease, early impactful decisions are often based on experience of referral centres.

We report the re-organisation which our institute (IEO, European Institute of Oncology), a cancer referral centre in Lombardy, went through to make our breast-imaging division pandemic-proof. Using personal-protective-equipment and innovative protocols, we provided essential breast-imaging procedures during COVID-19 pandemic without compromising cancer outcomes.

The emergency management and infection-control-measures implemented in our division protected both the patients and the staff, making this experience useful for other radiology departments dealing with the pandemic.

## Introduction

Since the severe acute respiratory syndrome coronavirus 2 (SARS-CoV-2) was first identified in Wuhan (Huebei Province, China) in December 2019,^1^ it has spread globally resulting in the ongoing coronavirus pandemic. As of 3 June 2020, more than 6.38 million cases of patients with such novel coronavirus disease (COVID-19) had been reported in more than 188 countries and territories, resulting in more than 380,000 deaths and more than 2.73 million hospitalisation.^2^ Until 3 June 2020, Italy had currently 39,893 active cases, one of the highest in the world.^2^ Overall, at the time of writing, there have been 233,515 confirmed cases and 33,530 deaths (a rate of 555 deaths per million population).^3^ However, due to the limited number of tests performed, the real number of infected people in Italy, as in other countries, was estimated to be higher than the official count.^4^ Lombardy, an area of 23,844 square kilometres (9,206 sq. mi) with 10 million people, is the most populous, the richest and most productive region in the country and one of the top regions in Europe under the same criteria,^5^ and has 89,205 confirmed cases and 16,145 deaths of COVID-19, representing, as on 3 June 2020, the most hard-hit part in all Italy, and probably all over the world.^6^

We discuss the re-organisation at an unprecedented scale of a breast-imaging division of our institute IEO, European Institute of Oncology), a cancer referral centre with high patient volume, located in Lombardy.

As the most common manifestation of COVID-19 is pneumonia,^1^ radiology units have been directly involved from the beginning of this emergency providing lung imaging assessment. At the same time, diagnostic imaging facilities must maintain in all phases of such pandemic, the standard radiologic support for cancer patients, including patients who needed to execute regular follow-up.^7^ To guarantee the appropriate safety standard, dedicated procedures are required to protect both the staff members and patients. Therefore, a reconfiguration of radiology units with the application of strict infection control procedures is essential as well as the establishment of protocols to manage subjects with suspected COVID-19 infection.

## Discussion

Italy was the first European nation adopting strict lockdown measures with the so-called “Phase 1” starting on 8 March and which have been in force for 55 days. Italy entered the so-called “Phase 2” of the COVID-19 emergency on 4 May 2020, with the start of the gradual lifting of the lockdown measures.^8^

With more than 65,000 breast imaging examinations and procedures provided in 2019, our breast-imaging division is one of the most important referral centre in Europe for breast cancer care.^9,10^ From the beginning of COVID-19 crisis, our goal was to continue to provide optimal care to breast cancer patients while reducing infective risk to patients and to staff.

**Figure 1.**
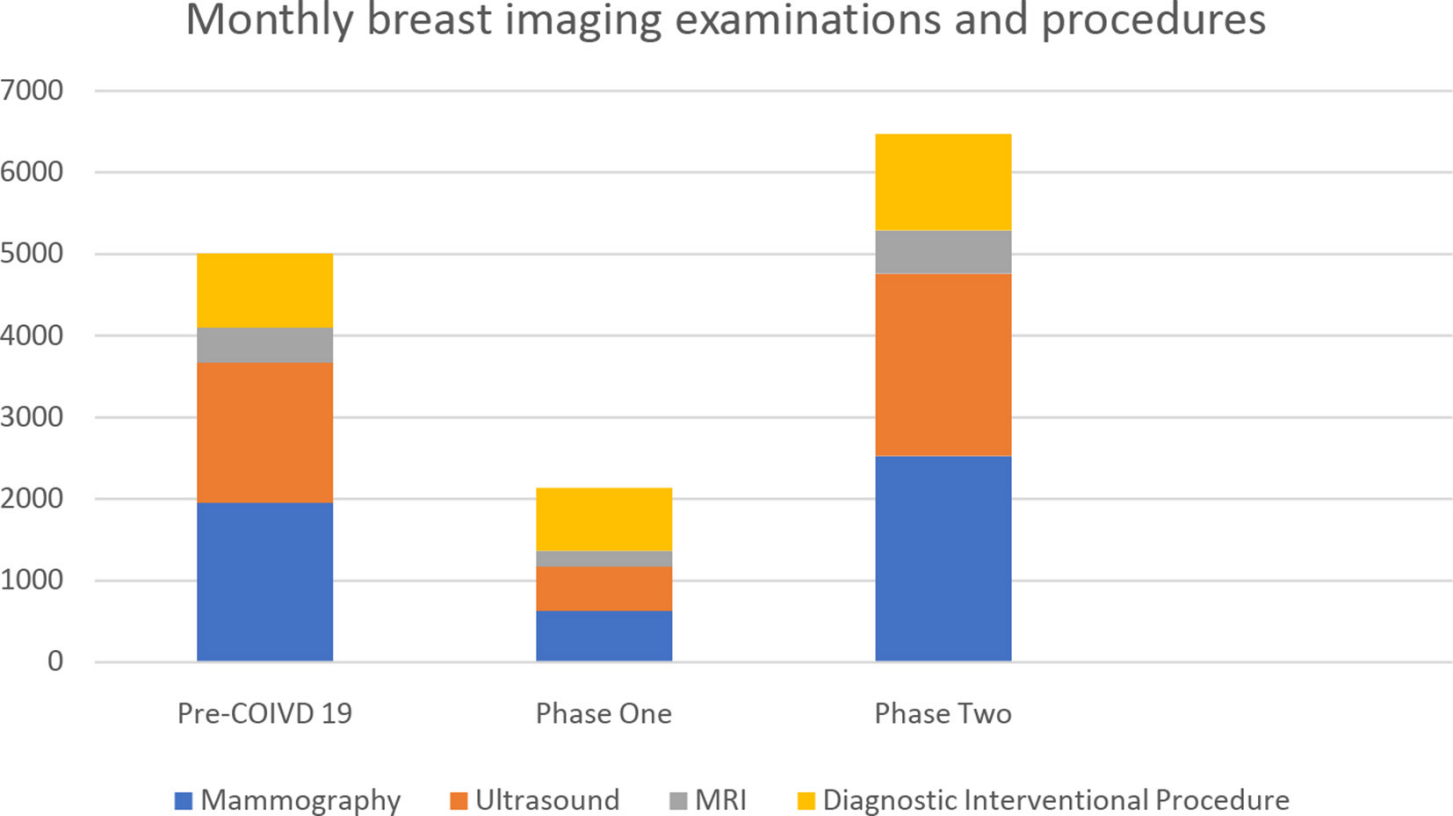
Distribution of breast-imaging examinations and diagnostic interventional procedures by month in a period before COVID-19 and during Phase 1 and Phase 2 in our institute.

During Phase 1, Lombardy’s government made a differentiation between elective and non-deferrable examinations/procedures to optimize the available healthcare resources, defining which patients could be treated, namely only emergency and oncologic patients.^11^ Accordingly, we stopped routine mammography and ultrasound screenings, as well as MRI examinations which were deferrable, but did not cancel appointments for patients with time-sensitive imaging needs. Specifically, we reported a decrease of 68% of both mammography and ultrasound (from 1950/month to 625/month and from 1720/month to 544/month, respectively) and of 52% of breast MRI (from 430/month to 195/month) (Figure 1).

Most of the interventional diagnostic procedures (such percutaneous ultrasound, stereotactic and MRI-guided biopsies) continued as normal in our centre, with a reported decrease of only 15% (from 910/month to 772/month) during Phase 1 (Figure 1).

Although deciding which patients deserve an examination is not always dependent on a list of predefined criteria, we are a team of 15 breast-dedicated radiologists and, in such extreme circumstances, we had to trust our expertise to select which patients to see, approaching this challenging task with diligence and vigilance. The main principle to decide whether to post-pone an off-patients’ examination is to find the right balance between risks (namely, a delay of diagnosis and treatment) and advantages (namely, to avoid being infected or to infect in-patients). Accordingly, our team of radiologists evaluated day-by-day the clinical history of all patients who had a booking in our unit: radiologists examined patient’s medical records or, when there were not available, directly contacted patients by phone. Generally, we strongly suggested to patients with flu-like symptoms, even if mild, and with immunodeficiency disorders to delay their appointments. On the other hand, the parameters that were considered for not post-to maintaining the scheduled examination appointment, as COVID-19 had shown to be more lethal in the elderly, their familiarity for breast cancer, their current treatment (*e.g.* immunotherapy) and the long time elapsed since the last breast-imaging examination.

Accordingly, in Phase 1 of COVID-19 pandemic we have made no significant changes in our diagnostic procedure workflows although we re-organised our department workflow to limit the risk of transmission between patients, and between patients and staff.

Radiologists, technical radiologists and nurses were trained to consider all the patients as potentially COVID-19 positive regardless of their symptoms and they underwent training for proper donning/doffing of PPE, namely hair cap, goggles for eye protection, disposable long sleeve fluid-resistant gown, disposable gloves, with coverage over gown cuffs, and a filtering face piece mask (FFP2) over goggles.^12^ Since COVID-19 is demonstrated to transmit also by touching contaminated surfaces/items, after each exam, the ultrasound probes and the mammography machine are cleaned with 1000 mg l^−1^ chlorine-containing disinfectant.^13^ Moreover, our unit obtained a backup call team to serve as the “clean team” for grabbing supplies and taking over after a procedure on a COVID positive/under investigation patient.

As mentioned above, during Phase 1, the number of radiological examinations decreased, due to both the cancellation of non-urgent visits and the fear of the population of visiting hospitals, which were considered a potential source of contagion. With fewer patients to see, radiologists and residents changed their work routines, using work time to make progress on research topics or to participate in webinars or to learn about trending topics. Such research activities were not formally restructured, and no tracking was implemented because the managers of our unit fully respected the personal way of dealing with such troubled and unusual times. Despite a lack of formal monitoring, our team showed a great sense of responsibility and all radiologists, when not engaged in clinical activity, devoted themselves to the study and/or to research activities with great seriousness, compatibly with the different predisposition to research and their personal impediments. Finally, some breast-imaging employees also have volunteered to be deployed to other areas if they have the necessary skills and experience. During Phase 1, 4 out of 25 radiologists and around 25% of staff (both radiologists and other healthcare employees) of our division, especially those with young children, decided to go on a 2-weeks paid leave, funded in part by our institute and in part by the Government. Unfortunately, remote working was not possible due to both the nature of our job (which includes performing ultrasound exams or communicating bad news to oncological patients), and the technical limitations of our institute which does not have the appropriate technology to allow performance from home of some of our activities (*e.g.* reading of mammography or MRI).

In Phase 2, Lombardy government allowed our centre to open again to elective (*i.e.* deferrable) examinations, including the follow-up imaging exams which are crucial for breast cancer patients.^14^ Therefore, each radiologist, with the help of administration staff, is contacting the patients that were postponed, selecting which patients to see first in accordane with the criteria listed above, and all the staff is planning to work extended (from 8 am until 7 pm instead of 4 pm) and weekend hours (one Saturday every 3 weeks for each radiologist) to address the growing backlog of patients. We expect to recover all the patients we previously postponed within 3 months, in order not to defer the breast cancer patients’ follow-up beyond 90 days from their expected date. So far, the volume of patients in the first 2 months of Phase 2 is estimated to be around 30% more (from 10,020 to 12,950 a total of diagnostic and interventional procedures) than the 2-months before of the outbreak of COVID-19 in Italy, namely January and February (Figure 1).

Additional protections for radiologists and other staff members are continually enforced: all staff members and all the patients now must wear masks (surgical mask or FFP2) inside our centre. The same PPE used in Phase 1 are used by radiologists when they cannot guarantee the 1-metre distance from the patient for more than 15 min. This essentially mean that that hair cap, goggles for eye protection, disposable long sleeve fluid-resistant gown, gloves and FFP2 mask are still mandatory for breast ultrasound.

All machines (mammography and MR scans) and their parts (ultrasound probes) are still cleaned by radiologist technician with 1000 mg l^−1^ chlorine-containing disinfectant^13^ after each examination.

In addition, facilities have added social distancing measures, such as limiting the number of chairs in waiting rooms, allowing access only to patients (companions can only access in case of real need), and scheduling appointments 30 min apart or on alternate days.

While in Phase 1, outpatients were screened for COVID-19 through questionnaires and temperature checks before entering our centre, temperature of patients and staff are now screened twice: first in the lobby and again at the breast imaging front desk, in both cases by a professional health worker. All patients are informed that, in the presence of fever, cough and/or flu-like symptoms, one medical doctor of our institute will evaluate the possibility to admit or refuse patients.

Finally, all in-patients undergo the reverse transcription polymerase chain reaction test performed on respiratory samples obtained by a nasopharyngeal swab the day before their hospitalisation. The ultimate aim at our centre is to test even asymptomatic outpatients: although our healthcare system cannot currently afford such amount of testing, our centre uses its own founds to reach this result, as it seems to be the most effective way to provide the appropriate safety standard for cancer patients.^15^

In conclusion, how radiology divisions respond to any infectious disease outbreak is determined primarily by the estimated risk of cross-infection to the staff and other patients.^7^ When the risk is high, as in the current case of COVID-19 infection, strict control infection protocols need to be applied to reduce the spread of the disease. Notably, in our breast imaging division, no incidents between non-infected and infected patients have been documented so far, and there has been only few cases of COVID-19 infection of healthcare workers in our department. By sharing our experience, with the reconfiguration of our breast-imaging division in a cancer referral centre located in one of the most important outbreak of COVID-19 in the world, we offer a roadmap for proceeding and we aim to mobilise the global research community to generate the data that are critically needed to offer the best possible care to breast cancer patients in this pandemic and during potential future emergencies of this kind.
